# Control Strategies of Clubroot Disease Caused by *Plasmodiophora brassicae*

**DOI:** 10.3390/microorganisms10030620

**Published:** 2022-03-14

**Authors:** Christine Struck, Stefanie Rüsch, Becke Strehlow

**Affiliations:** 1 Faculty of Agricultural and Environmental Sciences, University of Rostock, Satower Str. 48, 18059 Rostock, Germany; stefanie.ruesch@gmx.de; 2 Faculty of Agriculture and Food Sciences, University of Applied Sciences Neubrandenburg, Brodaer Str. 2, 17033 Neubrandenburg, Germany; strehlow@hs-nb.de

**Keywords:** Brassicaceae, biological control, canola, clubroot, lime fertilizer, oilseed rape, soilborne disease

## Abstract

The clubroot disease caused by the soil-borne pathogen *Plasmodiophora brassicae* is one of the most important diseases of cruciferous crops worldwide. As with many plant pathogens, the spread is closely related to the cultivation of suitable host plants. In addition, temperature and water availability are crucial determinants for the occurrence and reproduction of clubroot disease. Current global changes are contributing to the widespread incidence of clubroot disease. On the one hand, global trade and high prices are leading to an increase in the cultivation of the host plant rapeseed worldwide. On the other hand, climate change is improving the living conditions of the pathogen *P. brassicae* in temperate climates and leading to its increased occurrence. Well-known ways to control efficiently this disease include arable farming strategies: growing host plants in wide crop rotations, liming the contaminated soils, and using resistant host plants. Since chemical control of the clubroot disease is not possible or not ecologically compatible, more and more alternative control options are being investigated. In this review, we address the challenges for its control, with a focus on biological control options.

## 1. Introduction

Clubroot caused by the soil-living, obligate biotrophic protist *Plasmodiophora brassicae* Woronin belongs to the most devastating diseases of cruciferous crops worldwide [1,2]. As Plasmodiophoridae the pathogen belongs to the Rhizaria—a group of protists [3,4]. Together with the protist groups Stramenopiles (also called Heterokonta) and Alveolata, the Rhizaria represent the eukaryotic supergroup SAR, which is a highly diverse group of eukaryotic organisms [5]. Clubroot causes root tumors which lead to the disruption of water and nutrient uptake. As a consequence, infection can result in wilting and stunting. Yield losses of oilseed rape from 10% yield reduction to a total yield loss occur including plant losses and reduced seeds per pod [6]. The development of disease and cellular changes of host plants after infection were recently described in detail by [7,8].

Successful management of the disease is difficult as chemical control of clubroot is not allowed or not successful. In soil, the pathogen survives as extremely robust, thick-walled resting spores. Those spores can be easily transported from field to field via infested soil on machinery, by animals, water, or wind [9]. In this way, the spread of the disease can occur rapidly within a region. Spreading over continents already took place very early on. In the 19th century, clubroot was first described in Russia [10]. It is assumed that clubroot arrived with immigrants, colonization movement, and early settlers from Europe to North America [7], South America [11], and Australia [12], who probably carried the pathogen with infested food and fodder or to which contaminated soil adhered. At present, clubroot is reported from all continents (except Antarctica) and more than 75 countries [13]. It is very likely that the disease is distributed throughout the world wherever cruciferous crops are grown or cruciferous vegetation is available. Regions where the pathogen occurs with high population densities are mainly humid, temperate areas [14]. The expansion of cropping areas and proximal crop rotation contribute to an increase in clubroot [1].

An important factor in clubroot management is the deployment of effective host plant resistance. Not all known R genes are active against all races and resistance can be eroded by new pathogen races. In this review, we do not address the details of resistance to clubroot; instead, we want to focus on the control options available in agricultural practice. Crop management practices are widely used to combat the disease. However, control measures such as crop rotation and raising soil pH are not enough in reducing the disease. To improve control strategies, in recent years, research on biological control measures has emerged as an increasingly important role in clubroot management. In addition, strengthening of the plants by plant growth stimulants has gained increasing significance.

In this review, we summarize the different options for combating clubroot, focusing on biological control.

## 2. Environmental Parameters Influencing *Plasmodiophora brassicae* Development

If clubroot host plants are repeatedly grown in the field, the resting spores of the pathogen can accumulate in the soil [15]. Those spores are robust and can persist in the soil for many years, leading to an infestation of cabbage plants even after several years of cultivation [14]. Wallenhammar [16] calculated that the level of infestation in the field only decreases below the detection level after 17.3 years. However, a more than 2-year cultivation break and a diverse rotation pattern can reduce resting spores in soil [17,18].

In order to combat the pathogen *P. brassicae*, knowledge of the optimal environmental conditions that lead to an outbreak of the disease is a prerequisite. The causal agent of clubroot disease *P. brassicae* was described in 1878 by Woronin [10]; and more than 50 years later its lifecycle was shown for the first time [19]. Since then, the environmental conditions that are optimal for the disease and by which it is promoted have been studied in detail (for a review see: [14,20]).

Temperature and soil moisture play important roles in the development of the disease. The strongest infection rate and symptom severity occur at 21–25 °C [21,22,23]. At temperatures significantly below 20 °C clubroot severity is considerably reduced [21,22,23]. High soil moisture, especially during the first two weeks after inoculation, or during the first and second infections, is necessary for the successful development of the disease [20,21,24]. Moreover, wet soil conditions favor the spread of motile zoospores [25]. The soil type has a weak influence on the infestation intensities. However, it was shown that sandy soils and soils with low humic content are suppressive to disease [26] and that clubroot severity was lower in sand than in loamy soils or clay [16,27,28].

Another important factor for clubroot development is the soil pH. A low pH value (pH 5 to <7) in the soil promotes spore germination [29,30] and usually results in a more severe infestation. As the pH value increases, from about 7.2 onwards, there is hardly any new infection in most cases [31,32,33].

In conclusion, the most conducive conditions for clubroot development are high summer temperatures combined with light acidic soils and good soil moisture during the first weeks after sowing. It can be assumed that this will have considerable regional and between-year effects in the occurrence of clubroot disease.

## 3. Agricultural Practices

In general, chemical control of soil-borne diseases is difficult and cost intensive and is also not allowed in many countries because of the ecological impact. Of the currently worldwide registered chemicals, the oomycete fungicides fluazinam and cyazofamid reduce clubroot [34,35]. They are registered for cabbage crops in some countries, but they are not allowed in EU countries for clubroot control. The use of these agents is difficult and expensive because they have to be drenched into the soil to be effective against clubroot. Therefore, appropriate crop management practices are important measures for clubroot control.

### 3.1. Plant Resistance

Sowing of resistant host plants is an effective way to suppress the disease. Several resistant loci have been identified by quantitative trait locus mapping in *Brassica napus* and *B. rapa* (for a review see: [36,37,38]). The most widely used resistance loci originate from *B. rapa*; however, these loci do not confer resistance to all *P. brassicae* pathotypes or, even more alarming, pathotypes can overcome the resistance. Therefore, resistance breeding remains an important tool to combat *P. brassicae* [7,39].

### 3.2. Crop Rotation and Tillage

Due to the high market prices of rapeseed, the area under cultivation has increased and the crop rotation has narrowed [40]. This led to increasing problems with the pathogen and, therefore, another important factor in the control of clubroot is the use of crop rotation. More than 2 years’ break or diverse crop rotations can reduce severe clubroot epidemics [15,40]. Moreover, also the preceding crop might have an influence on the clubroot infection. A recent study compared different preceding crops before oilseed rape was planted. The results yielded a 40 and 50% reduction in clubroot disease index and incidence rate, respectively, when soybean was planted before oilseed rape [18]. In addition, microbiome analyses of soil showed that within the soybean—oilseed rape soil—more bacteria and fungi with known biocontrol functions were detectable than in maize—or rice—oilseed rape soil [18]. It has recently been shown that resting spores are highly sensitive to UV light [41]. Therefore, tillage measures, that bring resting spores to the soil surface for exposure to sunlight, could be an effective way to support clubroot management.

### 3.3. Field Sanitation

An aspect often overseen by practitioners is that resting spores are very easily carried with soil particles, e.g., with machinery, with boots, with surface water, or with animals, thus, spreading the disease. Furthermore, it has also been shown that resting spores are transported by wind-borne dust or soil erosion from field to field [42]. Therefore, activities that can reduce the transport of spores from field to field as sanitization of farmers’ machinery or measures that help prevent soil erosion thus prevent initial infestation of fields [43,44].

Once the spores are in the field, they are propagated not only by cruciferous crops, also cruciferous weeds, catch crops and volunteer canola/oilseed rape serve as alternative host plants [45] that can be infected with clubroot at any time and should therefore be removed in a timely manner [43,46].

### 3.4. Soil pH

In addition, pH management by liming is an efficient way of controlling clubroot disease. Although liming is traditionally used as a control measure, it has been discussed as highly controversial [47,48,49]. One reason is that the term “liming” means the application of different formulations of lime. Mostly lime with varying proportions of calcium carbonate (often mixed with Mg^2+^) [50,51] cyanamide [33,48,52] is used. Moreover, a combination of calcium carbonate and calcium sulfate [53] or calcium hydroxide [48] is applied. Only rarely has calcium oxide, also called burnt lime or quicklime, been applied [12,54]. Few studies have attempted to compare different types of lime [26]. Besides the calcium concentration, other factors such as the amount of lime, date of application, and soil pH may have effects on clubroot development. However, Niwa et al. [55] showed that it is not the calcium effect that is decisive but rather the soil pH. Germination of the resting spores is drastically reduced by a neutral soil pH. Because of the diversity of substances and soil differences, comparability of most field trials or greenhouse studies exploring the effect of lime on clubroot is difficult, and therefore, results are highly diverse.

With the aim of recognizing the effect of different lime fertilizers on clubroot development in oilseed rape, we conducted a greenhouse experiment with up to 37 one-week-old seedlings per lime type under strictly controlled conditions as described in [6]. We used a variety of lime fertilizers commonly applied in practice (Table 1). Before planting, the substrate was treated with the lime formulations. Rough-grained lime materials were finely crushed and passed through a 2 mm-mesh sieve before being added. Calcium oxide was added at a rate of 10 tonnes CaO ha^−1^, all other fertilizers at 13 tonnes ha^−1^. The *P. brassicae* isolates were sampled on heavily infested fields in North Germany and propagated in susceptible oilseed rape cultivar Avatar (Norddeutsche Pflanzenzucht Hans-Georg Lembke KG) as described in [6]. We used three isolates from different fields in Northern Germany to test the liming effect on a broader basis. Resting spores were isolated from homogenized clubbed roots and spore concentration was estimated microscopically with a hemocytometer after the spores were stained with Evan’s blue [56]. Spore concentration was adjusted to a value of 2 × 10^6^–10^7^ spores per ml. One milliliter of the suspension was placed directly to the seedling.

To determine soil pH, mixed soil samples (20 mL) were prepared from all pots of each variant weekly. To each soil sample, 50 mL CaCl_2_ (0.01 M) was added and mixed thoroughly. After 2 h at room temperature, the samples were filtered and the pH values were determined. Six to seven weeks after inoculation, roots were removed from the pots, gently washed, and the disease rating was recorded on a 4-classes scale from 0 to 3 and combined as the disease severity index (DSI) [6,57].

**Figure 1:**
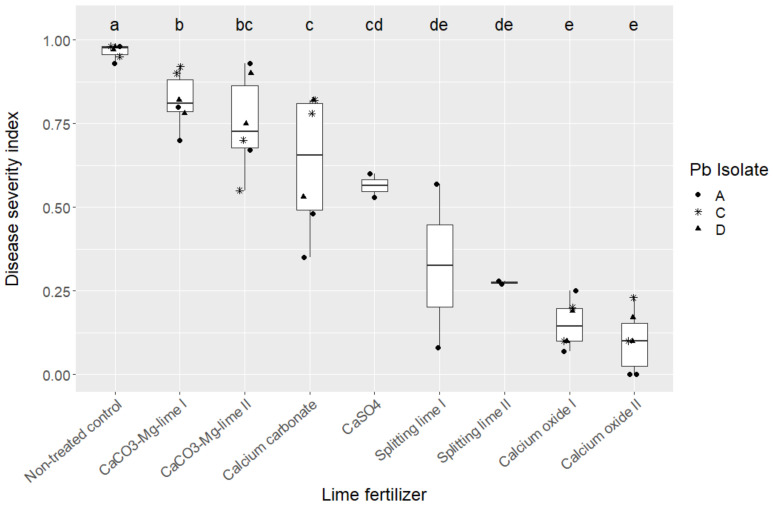
Effect of lime fertilizer on the disease severity index (DSI) of *Plasmodiophora brassicae* on the susceptible oilseed rape cv “Avatar”. Boxes and whiskers indicate interquartile ranges and subsequent 1.5-fold interquartile ranges. Different letters differ significantly at *p* < 0.05 according to Tukey’s honestly significant difference post hoc test on ranks of data.

Significant differences between the lime fertilizers on the development of clubroot were found by multiple comparisons of treatments by means using Tukey’s honestly significant difference post hoc test on ranks of data. Pearson’s correlation coefficient was used to test the association between DSI and pH value of the soil. Statistical analysis was performed using the software R version 3.6.1 (R core Team, 2019) and basic packages were complemented by the packages “agricolae” [58]. The results clearly show that the plants that grew in the most alkaline soil had the least clubroot damage (Figure 1, Table 2). Crucial is a soil pH > 0.68 during the seedling stage of the plants when they are particularly susceptible to infection. Due to the rapid reaction with water, calcium oxide reaches this pH value very quickly and thus contributes to the reduction in clubroot severity, while the other lime fertilizers do not raise the soil pH so early after application. A similar result was shown by Fox et al. [59], who used calcium hydroxide as lime, which is produced by the reaction of calcium oxide with water.

## 4. Clubroot Control Using Beneficial Microorganisms

The lack of effective control measures against *P. brassicae* makes it necessary to explore other, novel control options. The application of biological control measures could help to reduce soil-borne pathogens in particular. However, the complex life cycle of *P. brassicae* makes it difficult to apply biological control mechanisms against this pathogen. At least three phases can be used for control: (i) germination of the resting spores and/or secondary spores, which initiate (ii) primary infection of the root hairs and secondary infection of the root cortex; (iii) antagonism/competition against the developing pathogen within the host root tissue. In addition, resistance induction in host plants and changes in microbial communities in the rhizosphere soil could be biological control options [60].

Biocontrol agents that have been explored are bacteria or fungi including oomycetes. The mechanisms mostly are parasitism, antagonism by toxic/antibiotic secondary metabolites, and/or competition. Many studies have illustrated the biological control potential against soil fungi in *sensu stricto*. This refers to the direct antagonistic or inhibitory effect on the pathogen and not to an indirect effect such as plant growth promotion effect or induction of plant resistance [61,62].

Organisms such as, e.g., *Trichoderma* spp. and *Bacillus subtilis sensu lato*, are commercially employed in many control agents against a diverse group of plant pathogens [63,64,65,66,67]. There are numerous examples that illustrate that excellent control results can be achieved in in vitro trials [62,68]. Whereas in field trials, those successful control results often cannot be confirmed [69]. Therefore, for a successful control option, detailed research work must be preceded.

### 4.1. Antagonistic Bacteria

Bacteria of the *Bacillus subtilis* species complex are well studied for their biocontrol activity against plant pathogens. These bacteria have the potential to produce many hydrolytic enzymes and diverse secondary metabolites with antimicrobial properties [64,65]. One very well-characterized biological control agent patented strain in China is *B. subtilis* XF-1. Like other *Bacillus* strains, it produces fengycins, which are a group of nonribosomal lipopeptides. These metabolites have fungitoxic activities and are involved in the biocontrol effect of many *Bacillus* species (reviewed by [70,71]). Resting spores of *P. brassicae* directly treated with fengycins collapsed, and the cell contents leaked out [72]. Irrespective of this, the mode of action of fengycin was demonstrated with the *B. subtilis* strain NCD-2, which showed a reducing effect on clubroot; whereas, by using fengycin, defect mutants showed no effect against the clubroot pathogen [73]. In another experiment, Chinese cabbage seeds were soaked in a fengycin-producing *B. subtilis* XF-1 bacterial suspension and the bacterial culture disease incidence was reduced by 40% and 69%, respectively [74]. The authors demonstrated that the *B. subtilis* XF-1 treatment at an early stage of seedling development had the most positive effect. In the field, *B. subtilis* XF-1 reduced the disease index by about 17% [75]. We can therefore assume that in heavily infested fields, a reduction in this small amount is not useful to farmers.

The *B. amyloliquefaciens* strain QST713 (formerly *B. subtilis* strain) is registered for commercial uses (Serenade^®^) in many countries [76]. In Canada, it was tested against *P. brassicae*; however, in the field, the control success is limited [77]. Detailed greenhouse studies showed that the biofungicide Serenade^®^ applicated as soil drench reduced the clubroot disease incidence substantially [28,78,79]. Recently, besides a new strain of *B. amyloliquefaciens*, another member of the genus, *Bacillus*, *B. velezensis*, was described as a biocontrol agent against *P. brassicae* [68].

Several species of the bacterial genus *Lysobacter* are known for their activity against soil pathogens. These bacteria synthesize many hydrolytic enzymes and antimicrobial compounds and several commercial preparations are available against soilborne fungal pathogens [80]. By screening bacterial strains from vegetable rhizosphere soil [81], *Lysobacter antibioticus* strains were isolated whose culture filtrates reduced clubroot severity on Chinese cabbage after application as a soil drench or seed treatment. Another *Streptomyces* strain, *S. platensis* 3–10, was used to optimize the culture medium and reached an inhibition of resting spore germination up to 80% [82]. Recently, a strain of *Bacillus cereus*, MZ-12, isolated from the rhizosphere soil of symptomless *B. campestris* (pak choi) showed an inhibitory effect on germination of resting spores. Co-inoculation of the pak choi plants with *P. brassicae* spores and MZ-12 resulted in a 64% reduction in clubroot gall formation [83].

It has been shown repeatedly that the results of in vitro and greenhouse experiments cannot be achieved with field experiments. For example, the bacterial strain *Zhihengliuella aestuarii* B18 isolated from rhizosphere of *Brassica juncea* showed a control efficiency of 63.4% against clubroot in greenhouse tests, whereas the control effect in the field was only 49.7% [84]

In addition to free-living microorganisms in the rhizosphere or epiphytic-living microorganisms, endophytic-living microorganisms can also contribute to biological control. Ahmed et al. [85] provide an overview of research carried out with endophytic bacteria and fungi as biocontrol agents. Mostly, endophytic bacteria derived from the rhizosphere enter the plant and colonize the plant tissue without any negative effect on the plant [86]. In many cases, this form of bacterial colonization contributes to the promotion of plant growth by different mechanisms [87]. However, the antagonistic activity by endophytic actinobacteria against clubroot has been reported by Lee et al. [88]. They isolated 81 actinobacterial strains from the surface-sterilized root tissue of Chinese cabbage. Among them, they selected three strains that showed in vivo biocontrol activities against *P. brassicae*. Two of these strains were identified as *Microbispora rosea*, the third strain as *Streptomyces olivochromogenes* [88]. Wang et al. [89] tested 63 actinobacteria strains isolated from the rhizosphere of Chinese cabbage by measuring the inhibition of the germination of *P. brassicae* resting spores. This resulted in six strains that were used in greenhouse and field trials against clubroot. The strain A316 showed high control values of 73.69% in a glasshouse experiment and 65.91% in a field experiment [89].

A recent paper by Wei et al. [90] presents a detailed analysis of bacterial metabolites with biocontrol functions against clubroot. The work shows that co-culturing bacterial species of different genera produces more relevant metabolites than culturing bacterial species of the same genera. The results reveal that bacterial interactions between genera promote the production of biocontrol active substances.

Increasingly, microbiome studies of the rhizosphere are showing that the microbial communities are complex and highly variable [87]. Recently, a study was presented comparing heavily *P. brassicae*-contaminated soils with weakly contaminated soils. The results showed that the bacterial communities of both soil types differed significantly [91]. Furthermore, the study showed that certain groups of bacteria were mainly found in weakly contaminated soils [91]. Such studies suggest that individual bacterial species cannot generally act as control organisms. Instead, in each soil type, the composition of microbial communities differs and one must assume and consider, for further work, that groups of organisms act together.

### 4.2. Antagonistic Fungi

The soil-borne ascomycetous fungus *Phoma glomerata* (current name: *Didymella glomerata*) has been described as a plant pathogen [92,93] as well as a potential biocontrol agent [94,95]. The strain *P. glomerata* no. 324 produces the secondary metabolite epoxydon. Although this substance showed very weak antifungal activity, it reduced clubroot symptoms on Chinese cabbage after spraying over the infested soil (30 mL extract solution containing 250 µg mL^−1^ per 180 mL pot) completely [96]. However, this work was conducted in the 1990s and has never been commercialized.

The fungus *Clonostachys rosea* f. sp. *catenulata* (syn. *Gliocladium catenulatum*) is widely known as a biocontrol agent. In many countries, it is commercialized as the biofungicide Prestop^®^ with control activity against several soil-borne plant pathogens in various crops [97]. Neither the fungus *C. rosea* nor the biofungicide Prestop^®^ had any effect on the germination and viability of the resting spores of *P. brassicae* [98]. However, soil drench treatments on *B. napus* seedlings 7 to 14 days after seeding resulted in a reduction in clubroot severity of about 90%. The fungus colonized the plant root system and, in this way, suppressed clubroot. In addition, it appeared to induce plant resistance since some induced plant resistance-associated genes were up-regulated [98].

The fungal genus *Trichoderma* comprises several species that are well studied as biological control agents against various plant pathogens [66,67]. In greenhouse pot experiments, the control efficiency of *T. harzianum* strain T4 against *P. brassicae* in Chinese cabbage was about 79% [99]. Another work showed the control effect of *T. harzianum* strain LTR-2 in Chinese cabbage in the field. The disease incidence was lowered from 96.7% (untreated control) to 51.3% (seeds treated with spores of *T. harzianum* LTR-2) [100].

Endophytic (mutualistic) fungi grow within their host plants tissue without causing visible disease symptoms [101]. They may have beneficial effects on the plant via plant growth promotion or by suppressing plant pathogens or pests [102,103]. The ascomycetous soil fungus *Heteroconium chaetospira* has been isolated from cabbage roots and was described as an endophytic root fungus growing throughout the cortical cells [104,105]. In greenhouse experiments, *H. chaetospira* reduced clubroot in Chinese cabbage plants by 90 to 100% after inoculation with low to moderate *P. brassicae* resting spore concentrations (up to 105 spores per g of soil). Severely diseased plants after inoculation with 10^6^ spores per g of soil could not be protected by the endophytic fungus [106]. In field experiments, the disease reduction was lower; however, there was no reduction effect at high soil moisture [106]. Further investigations showed that *H. chaetospira* induced resistance reactions in *Brassica napus* plants. The phenyalanine ammonia lyase (PAL) activity, which was increased by *H. chaetospira*, and the upregulated transcript levels of several genes known to be involved in inducing plant resistance (ethylene/jasmonic acid synthesis, PR-2 protein, auxin biosynthesis) served as indicators for the resistance reaction [107]. Another endophytic fungal genus associated with plant roots is *Acremonium*. The species *A. alternatum* can colonize root cells of Brassicaceae. When Arabidopsis plants were co-inoculated with *A. alternatum* and *P. brassicae*, the disease index of clubroot was reduced by up to 50% [108].

## 5. Conclusions

Clubroot management has always been a challenge for farmers and chemical as well as classical agronomic measures have not been fully successful. In the last 10 to 20 years, a great wealth of work has been carried out on the biological control of clubroot. Some of the tested biofungicides appear promising for controlling the pathogen. However, to date, none of these approaches has become established in practice. Several studies showed that under a high disease pressure, the control activity of biocontrol agents is too weak [77,106]. Nevertheless, it is important to pursue these efforts further. There are a number of microbial control agents against clubroot, but our knowledge of the modes of action is too low (competition, hyperparasitism, antibiosis, mixed modes with induced plant resistance?). Research into the mode of action is required to bring more agents to the approval stage. However, even more complex is the nature of soil microbial interactions and the role of microorganisms of the rhizosphere for plant health is still wildly unknown [109]. The host plant-associated microbiomes of the rhizosphere affect the development of soil pathogens and the diseases they transmit. In the past, this interaction has often been neglected in research. Instead, the focus was on individual pathogens and their host plants in their particular environment [110]. Recently, the number of studies on the effect of soil microbiome has increased [111]. Raaijmakers and Mazzola [112] discussed that the functional similarity of immune-suppressed soils across many agroecosystems suggests that it may be possible to develop disease-suppressive soil microbiomes using a universal approach. Microbiome engineering is being vigorously discussed as the biocontrol method of the future [113]. It is becoming increasingly clear that the composition of the rhizosphere microbiome is important [114]. It has been shown that the bacterial diversity of the seed microbiome of oilseed rape differs depending on the cultivar [115]. Should there be less emphasis on pathogen control in the future and more focus on strengthening and compensating plants and influencing plant development by plant growth-promoting rhizobacteria? [116]. The composition of the microbiome could play an important role here. Lebreton et al. [117] showed that microorganism communities of healthy and clubroot-diseased plants differ considerably. Much attention should therefore be paid to comparing different rhizosphere microbiomes in order to identify important microorganisms. Can we compose a suitable microbiome? We are still far from an effective clubroot biological control option, but it is becoming possible that interactions of microbial communities could make a general contribution to the control of soil-borne plant diseases [118].

## Figures and Tables

**Table 1:** Characteristics of the lime fertilizers used.

Lime Fertilizer	Components (%)					CaO Equivalent (%)	pH of Soil (6 dpi)
	CaCO_3_	CaO	MgO	SO_3_	SO_4_		
Calcium carbonate	80	---	---	---	---	45	6.44
CaCO_3_-Mg-lime I	80	---	5	---	---	48	5.42
CaCO_3_-Mg-lime II	50	---	35	---	---	19	6.39
CaSO_4_	68	---	1–2	---	4.5	38	6.58
Splitting lime I ^1^	80 + 75	---	---	+25	---	22.5 + 22.5	6.71
Splitting lime II ^1^	80 + 75	---	---	+25	---	45 + 45	6.72
Calcium oxide I	---	38	3	---	---	38	7.00
Calcium oxide II	---	90	1	---	0.3	90	7.34
Non-treated control	---	---	---	---	---	---	4.90

^1^ additional application of SO_3_-containing lime 6 days after inoculation.

**Table 2:** Disease severity index (DSI). Standard deviation (SD), number of plants tested (N), infestation frequency (IF).

Lime Fertilizer	DSI	SD	N	IF (%)
Calcium carbonate	0.63	0.28	120	86.67
CaCO_3_-Mg-lime 1	0.75	0.24	120	95.83
CaCO_3_-Mg-lime 2	0.82	0.25	160	96.67
CaSO_4_	0.57	0.28	40	82.50
Splitting lime 1 ^1^	0.33	0.27	40	47.50
Splitting lime 2 ^1^	0.28	0.08	40	40.00
Calcium oxide 1	0.15	0.25	120	29.60
Calcium oxide 2	0.10	0.15	120	23.33
Non-treated control	0.97	0.08	120	100.00

^1^ Additional application of SO_3_-containing lime 6 days after inoculation.

## Data Availability

Not applicable.
